# Adverse event profiles of microscopic colitis in the Japanese Adverse Drug Event Report (JADER) database

**DOI:** 10.1038/s41598-022-22257-2

**Published:** 2022-10-21

**Authors:** Kaito Yamashiro, Mika Jouta, Kouichi Hosomi, Satoshi Yokoyama, Yuu Ozaki, Atsushi Hirata, Fumihiko Ogata, Takehiro Nakamura, Shigeharu Tanei, Naohito Kawasaki

**Affiliations:** 1 Laboratory of Public Health, Faculty of Pharmacy, Kindai University, 3–4–1, Kowakae, Higashi-Osaka, Osaka 577–8502 Japan; 2 Department of Pharmacy, Kindai University Nara Hospital, 1248–1, Otodacho, Ikoma, Nara 630–0293 Japan; 3 Division of Drug Informatics, Faculty of Pharmacy, Kindai University, 3–4–1 Kowakae, Higashi-Osaka, Osaka 577–8502 Japan; 4 Faculty of Pharmaceutical Sciences, Nihon Pharmaceutical University, 10281 Komuro, Ina-Machi, Kitaadachi-Gun, Saitama, 362–0806 Japan

**Keywords:** Diseases, Gastroenterology, Medical research, Risk factors

## Abstract

Microscopic colitis (MC) is a chronic inflammatory bowel disease that is characterized by nonbloody watery diarrhea. The epidemiology in Japan differs from that in Europe and the United States, but little information is available from epidemiological surveys of MC in Japan. This study aimed to provide a new hypothesis regarding the factors associated with MC by using the Japanese Adverse Drug Event Report (JADER) database. “Colitis microscopic” (preferred term code: 10056979) cases entered into the JADER database between 2004 and 2021 were analyzed. Of the 246,997 cases in the JADER database, 161 cases were observed to be associated with MC. A Weibull analysis revealed that the median onset duration of MC (interquartile range) was 72.5 (36.0‒125.5) days in lansoprazole users and 116.0 (60.3‒1089.0) days in aspirin users. A multiple logistic regression analysis revealed that MC was significantly associated with the female sex, as well as ages ≥ 60 years and drugs including lansoprazole, aspirin, and nicorandil. A subset analysis revealed that MC was positively associated with obesity in female cases. Our study cannot demonstrate a causal inference between MC and each drug; however, the findings suggest that MC was associated with nicorandil as well as with lansoprazole and aspirin.

## Introduction

Microscopic colitis (MC) is a chronic inflammatory bowel disease that is characterized by nonbloody watery diarrhea, with few or no endoscopic abnormalities^1^. There are two main subtypes of this disorder, including lymphocytic colitis (LC) and collagenous colitis (CC). The incidences of LC and CC have been reported to be 1.1‒5.2 and 3.1‒5.5 cases per 100,000 population in Europe and North America, respectively^2,3^. Clinical symptoms of LC and CC include chronic and persistent diarrhea, abdominal pain, weight loss and flatulence^3,4^, which can reduce the quality of life of patients^5^.

The reported incidence of MC is lower in Japan than in Europe and the United States, and CC is more common than LC in Japan^6^. In Europe and the United States, MC is typically associated with autoimmune disorders such as thyroiditis, celiac disease, and rheumatoid arthritis^7^. In contrast, of the Japanese patients who develop CC, the most common underlying diseases are hypertension and reflux esophagitis, with autoimmune disorders being rare^8^. As the situation of MC varies between Japan and other countries, further research is needed to identify the situation with regard to MC in Japan.

The underlying mechanisms of MC remain unclear, but an association has been suggested with abnormal reactions to luminal antigen, autoimmunity, bile acid malabsorption, infection, certain drugs and abnormal collagen metabolism^9^. The risk of MC has been found to be higher in females aged > 50 years, with a reported ratio of 4.4–7.9 in CC and 1.8–5.0 in LC compared with males^3,10–13^. Additionally, smoking and specific drugs have been reported to be risk factors for MC^14^, with drugs including proton pump inhibitors (PPIs), selective serotonin reuptake inhibitors (SSRIs), nonsteroid anti-inflammatory drugs (NSAIDs), antidepressants (such as carbamazepine and duloxetine), and β-blockers^15–17^. It is recommended to choose a first-line therapy for MC that avoids such risk factors. If there is no improvement in symptoms, loperamide, mesalazine, salazosulfapyridine, or oral steroids can be considered as treatments^18,19^. As the proportion of drug-induced cases of CC is considered to be higher in Japan than in Europe and the United States^6^, it is important to avoid risk factors for MC treatments; however, data regarding causative drugs are controversial^20^.

The spontaneous reporting system (SRS) is a valuable tool for evaluating drug-related adverse events^21^. In Japan, the Pharmaceuticals and Medical Devices Agency (PMDA) manages a SRS known as the Japanese Adverse Drug Event Report (JADER) database. Although the JADER database has several biases (such as a notoriety bias and competition bias) that may affect study outcomes^22,23^, it contains data regarding severe and rare adverse events, including drug-induced MC. Few reports have comprehensively evaluated the patient backgrounds of MC, causative drugs, or onset durations. Therefore, the purpose of this study was to provide a new hypothesis regarding the factors associated with the onset of MC by using the JADER database.

## Materials and methods

### Data source and data selection

**Figure 1:**
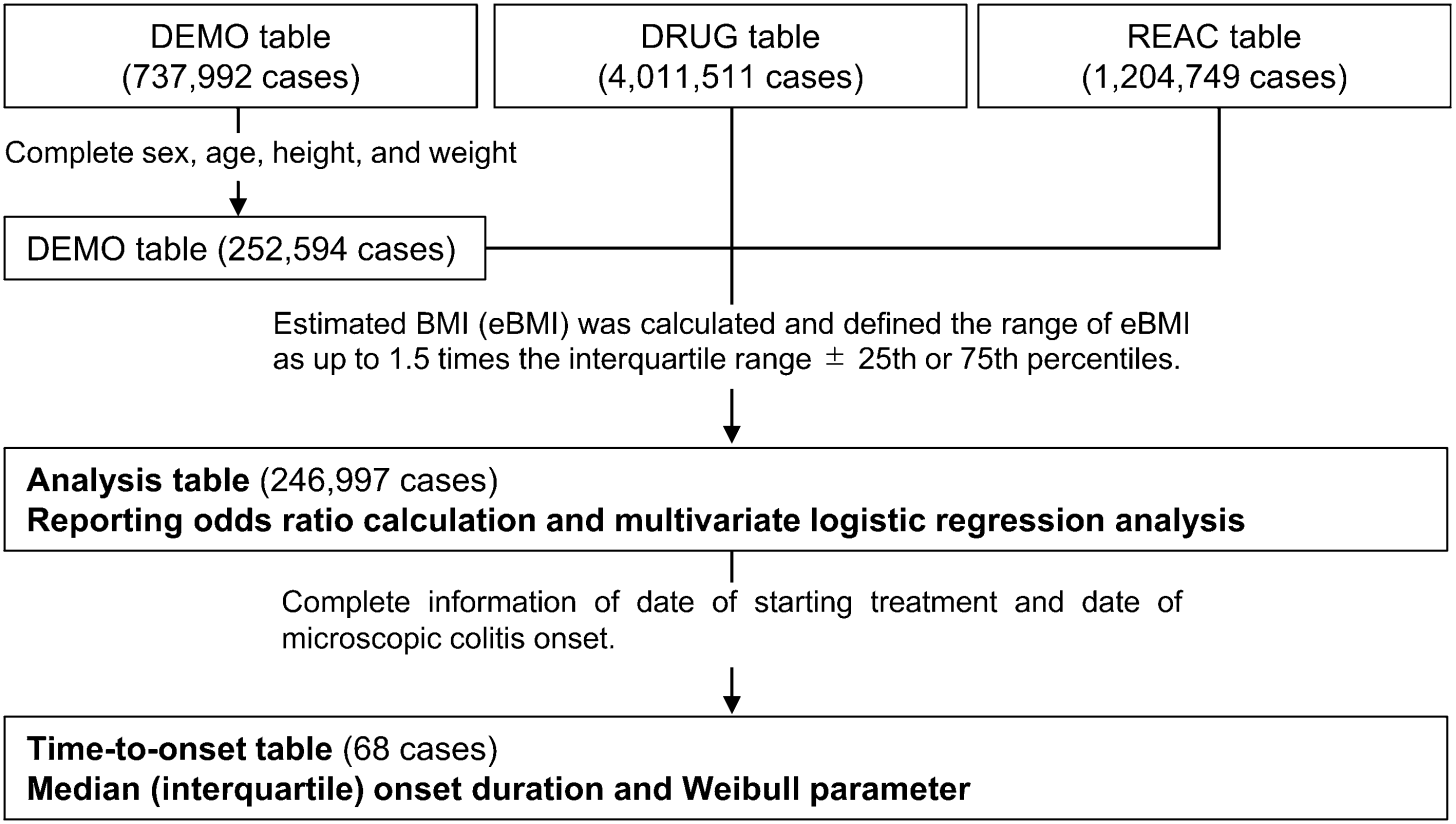
Flowchart of dataset construction from the Japanese Adverse Drug Event Report database.

The SRS adverse event data recorded in the JADER database between April 2004 and August 2021 were downloaded from the PMDA website (http://www.pmda.go.jp/). The database consists of four tables: patient background information (DEMO), drug information (DRUG), adverse reactions (REAC), and medical history (HIST). All of the tables (except for the HIST table) were connected by an ID number. A flowchart of the data selection from the JADER database is shown in Fig. 1. Patients with missing or unclear data on sex, age, height, or weight were excluded from all of the reports. Height and weight data are entered into the JADER database in the form of 10-cm-denominated ranges (height) and 10-kg-denominated ranges (weight). We used the intermediate values in these classifications of height and weight as the continuous variables. For example, for a patient with height and weight in the 160‒169 cm and 50‒59 kg ranges, respectively, the intermediate values of height and weight would be 165 cm and 55 kg, respectively. To evaluate body type, we calculated estimated BMI (eBMI) by using the following formula^24^, which is shown here for the example patient above:

$$eBMI\, = \,Intermediate \, value \, of \, weight \, \left( {kg} \right)/[Intermediate \, value \, of \, height \, \left( m \right)]^{2} \, = \,55 \, \left( {kg} \right)/\left[ {1.65 \, \left( m \right)} \right]^{2} \, = \,20.2 \,kg/m^{2}$$

We excluded outliers of eBMI by using the boxplot method^25^ and we defined the range of eBMI as up to 1.5 times the interquartile range ± 25th or 75th percentiles. eBMI was classified as follows: underweight, eBMI < 18.5 kg/m^2^; normal, 18.5 ≤ eBMI < 25.0 kg/m^2^; and obese, eBMI ≥ 25.0 kg/m^2^. After applying the exclusion criteria, 246,997 patients were included in the table, which was renamed as the “Analysis table”. The following age classifications were then applied to the table: < 10, 10‒19, 20‒29, 30‒39, 40‒49, 50‒59, 60‒69, 70‒79, 80‒89, 90‒99 and 100‒109 years. The World Health Organization (WHO) defines “elderly” as those individuals who are greater than 65-years old. Accordingly, we stratified the analysis table into two groups: 0‒59-years-old and ≥ 60-years-old. The data were further limited to those cases with complete information regarding the date of the start of drug administration and the date of onset of adverse events, and this table was named the “Time-to-onset table” (68 cases). Time-to-onset was defined as the period from the start of drug administration to the onset of MC. In this study, time to onset was defined as the shortest duration to onset. The drugs that were included in the analysis of time-to-onset were lansoprazole and aspirin, which were reported in > 10 cases and identified the in multivariate logistic regression analysis.

### Definitions of adverse events and drugs of interest

The definition of adverse events that was used in this study was that of the Medical Dictionary for Regulatory Activities/Japanese (MedDRA/J) version 24.0 (https://www.jmo.gr.jp/jmo/servlet/mdrLoginTop). We identified cases of MC based on the preferred term (PT) “Colitis microscopic” (PT code: 10056979) of the MedDRA/J. In the JADER database, a clinical outcome profile was entered into “Uncertain”, “Recovered”, “Improved”, “Unimproved”, “With sequelae”, and “Death”, and this outcome of MC was investigated. In patients with MC, drugs that were administered in at least five cases were considered drugs of interest, and these drugs are listed in Table 1. Among the drugs of interest, the most common therapeutic categories were defined as PPIs, NSAIDs, angiotensin converting enzyme inhibitors (ACE-Is)/angiotensin II receptor blockers (ARBs), statins, β-blockers, bisphosphonates, and calcium channel blockers.

**Table 1:** Groupings of the analyzed drugs according to frequency of use.

n	Drug
> 100	Lansoprazole
20‒100	Aspirin, amlodipine, furosemide
10‒19	Loxoprofen, nicorandil, allopurinol, magnesium oxide, candesartan, rosuvastatin, carvedilol, etizolam, olmesartan, alendronic acid, rebamipide, bisoprolol, clopidogrel, ferrous citrate, mecobalamin, diclofenac, valsartan, atorvastatin, brotizolam, teprenone, prednisolone
5‒9	Ursodeoxycholic acid, alfacalcidol, rabeprazole, pravastatin, nifedipine, cilostazol, warfarin, zolpidem, enalapril, mosapride, esomeprazole, celecoxib, losartan, diltiazem, benidipine, febuxostat, spironolactone, flunitrazepam, loperamide, vonoprazan, risedronic acid, isosorbide nitrate, apixaban, triazolam, albumin tannate, tocopherol nicotinate, famotidine

### Statistical analysis

The median and interquartile range (IQR) days to onset and Weibull shape parameters^26^ were used to clarify the time-to-onset profile for MC. The Weibull distribution includes a scale parameter (α) and a shape parameter (β). The shape parameter indicates the failure rate distribution over time and is categorized as follows: early-failure type involves the upper limit of the 95% confidence interval (95% CI) of β less than 1, and incidence may decrease over time; random-failure type involves 95% CI of β and includes 1, with the incidence possibly being constant over time; and the wear-out-failure type involves the lower limit of 95% CI of β greater than 1, and the incidence may increase over time.

**Figure 2:**
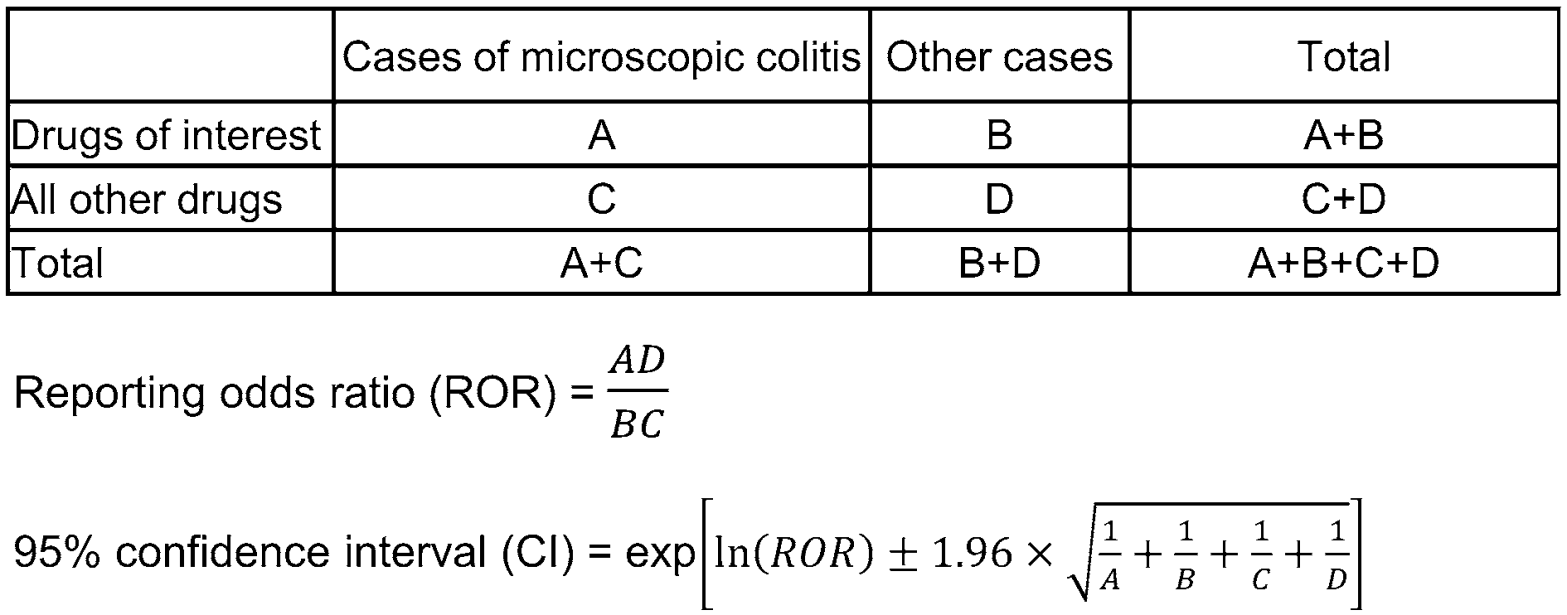
Two-by-two contingency table for calculating the reporting odds ratio and 95% confidence interval of microscopic colitis.

A two-by-two contingency table was compiled based on the presence or absence of MC and the drug of interest. The reporting odds ratio (ROR) and 95% CI were calculated to evaluate the safety of each drug based on a previous study^27^ (Fig. 2). A signal was considered to be positive when the lower limit of the 95% CI was greater than 1. We also performed a multiple logistic regression analysis to consider the confounding factors based on the methods that were used in previous studies^28,29^. Sex, age and eBMI are hypothesized to be confounding factors, and these factors were forced entry as the explanatory variables. To evaluate the effectiveness of all of the drugs of interest listed in Table 1 (except for albumin tannate, prednisolone, and loperamide), a forward and backward stepwise selection with a significance level of 0.05 was performed in all of the cases^29,30^. Albumin tannate, prednisolone, and loperamide were excluded from the stepwise selection because these drugs are used to treat MC. The objective variable was set to MC, and the explanatory variables were set to sex, age, eBMI and drugs which were selected using by the stepwise method. Additionally, a subset analysis was performed to evaluate the effect of sex. The explanatory variables for female and male patients were set to age, eBMI, lansoprazole, and aspirin, as these variables are reported to be associated with MC. The final multiple logistic regression model was as follows:

$$\log \left( {odds} \right)\, = \,\beta_{0} + \,\beta_{1} S\, + \,\beta_{2} A\, + \,\beta_{3} E\, + \,\beta_{4} L\, + \,\beta_{5} AS\, + \,\beta_{6} MO\, + \,\beta_{7} N\, + \,\beta_{8} D\, + \,\beta_{9} R\, + \,\beta_{10} F\, + \,\beta_{11} V\left( {in \, all \, cases} \right),$$

$$\log \left( {odds} \right)\, = \,\beta_{0} \, + \,\beta_{1} A\, + \,\beta_{2} E\, + \,\beta_{3} L\, + \,\beta_{4} AS\left( {in \, female \, and \, male \, cases} \right).$$

S: sex (female vs. male), A: age (≥ 60 years vs. < 60 years), E: eBMI (obese or underweight vs. normal), L: lansoprazole, AS: aspirin, MO: magnesium oxide, N: nicorandil, D: diclofenac, R: rabeprazole, F: flunitrazepam, and V: vonoprazan.

Multicollinearity diagnosis was performed by variance inflation factors (VIF). All VIF values of the variables in the final multiple logistic regression model were less than 2 (Supplementary Table S1). In this study, all of the statistical analyses were performed by using JMP Pro, version 15.0.0 (SAS Institute Inc., Cary, NC, USA). A p value less than 0.05 was considered to be statistically significant.

### Ethics approval statement

No ethical approval was required for this study.

## Results

There were 737,992 cases in the DEMO table, 4,011,511 cases in the DRUG table, and 1,204,749 cases in the REAC table of the JADER dataset. After excluding the missing data, 246,997 cases were analyzed, and 161 cases of MC were identified (47 males and 114 females; 13 individuals in those aged 0–59 years and 148 individuals in those aged ≥ 60 years). The mean ± standard deviation (minimum to maximum) of eBMI was 21.5 ± 3.9 (14.6–31.2) kg/m^2^ in MC cases and 21.8 ± 3.9 (11.3–31.2) kg/m^2^ in non-MC cases. When regarding the clinical outcome profile, the numbers of uncertain, recovered, improved and unimproved cases were 8, 77, 74 and 2 cases, respectively. Moreover, there were no cases with sequelae or death. Table 1 groups the analyzed drugs according to frequency of use. The top four reported drugs for MC (in descending order) were lansoprazole (n = 128 cases), aspirin (n = 46 cases), amlodipine (n = 28 cases), and furosemide (n = 21 cases). The number of cases and crude RORs of MC are summarized in Supplementary Tables S2 and S3 online.

**Figure 3:**
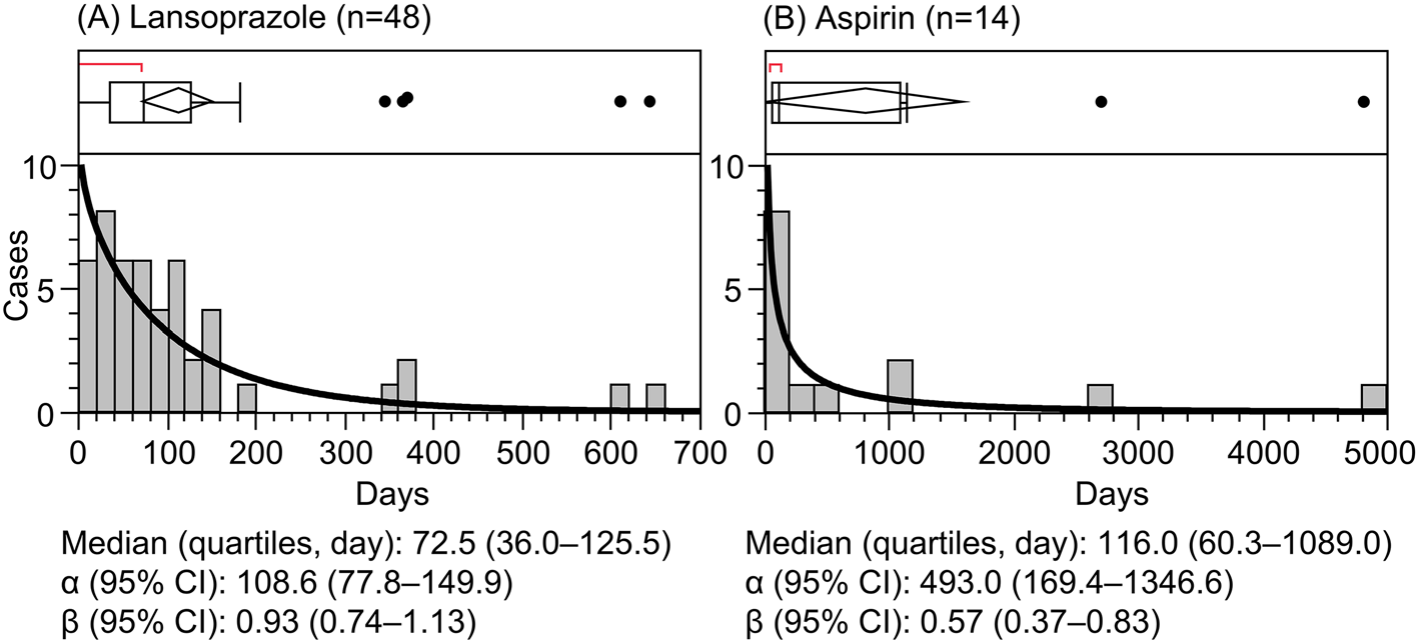
Histograms and Weibull shape parameters of microscopic colitis for (**A**) lansoprazole and (**B**) aspirin.

Figure 3 shows the median onset durations (IQRs) and Weibull parameters for lansoprazole and aspirin. Lansoprazole was used in 48 cases and aspirin in 14 cases. In the lansoprazole cases, the median onset duration (IQR) and the β parameter were 72.5 (36.0‒125.5) days and 0.93 (0.74‒1.13), respectively; in addition, the onset of MC was considered to be of the random-failure type. In the aspirin cases, the median onset duration (IQR) and the β parameter were 116.0 (60.3‒1089.0) days and 0.57 (0.37‒0.83), respectively, and the onset of MC was considered to be the early-failure type. Furthermore, the median onset duration of lansoprazole was shorter than that of aspirin.

Tables 2 and 3 show the results of the multiple logistic regression analysis of MC by using the variables of individual drugs and patient background. The multiple logistic regression analysis revealed that MC was significantly associated with female sex [adjusted ROR (aROR): 3.26, 95% CI: 2.31‒4.60], age ≥ 60 years (aROR: 3.94, 95% CI: 2.22‒6.98), and lansoprazole (aROR: 35.55, 95% CI: 23.92‒52.84), aspirin (aROR: 1.66, 95% CI: 1.14‒2.41), magnesium oxide (aROR: 0.38, 95% CI: 0.22‒0.64), nicorandil (aROR: 2.11, 95% CI: 1.19‒3.74), diclofenac (aROR: 2.21, 95% CI: 1.18‒4.11), rabeprazole (aROR: 2.26, 95% CI: 1.09‒4.65), flunitrazepam (aROR: 2.32, 95% CI: 1.01‒5.31) and vonoprazan (aROR: 3.41, 95% CI: 1.38‒8.44). In females, MC was significantly associated with age ≥ 60 years (aROR: 4.45, 95% CI: 2.16‒9.19), obesity (aROR: 1.58, 95% CI: 1.03‒2.43), lansoprazole (aROR: 45.33, 95% CI: 27.22‒75.48), and aspirin (aROR: 1.59, 95% CI: 1.02‒2.46). In males, MC was significantly associated with lansoprazole (aROR: 15.32, 95% CI: 8.12‒28.91) and aspirin (aROR: 2.80, 95% CI: 1.52‒5.14).

**Table 2:** Multiple logistic regression analysis of microscopic colitis using variables of drug and patient background in all cases.

	All cases			
	Cases n = 161	Non-cases n = 246,836	aROR (95% CI)	p value
Sex (female)	114	114,463	3.26 (2.31‒4.60)	< 0.001***
Age ≥ 60 years	148	164,117	3.94 (2.22‒6.98)	< 0.001***
eBMI				
Obese	36	44,574	1.21 (0.82‒1.78)	0.334
Underweight	28	40,153	1.17 (0.77‒1.79)	0.467
Normal	97	162,109	Ref	‒
Lansoprazole	128	22,387	35.55 (23.92‒52.84)	< 0.001***
Aspirin	46	20,578	1.66 (1.14‒2.41)	0.008**
Magnesium oxide	15	25,972	0.38 (0.22‒0.64)	< 0.001***
Nicorandil	15	4219	2.11 (1.19‒3.74)	0.011*
Diclofenac	11	5544	2.21 (1.18‒4.11)	0.013*
Rabeprazole	8	10,399	2.26 (1.09‒4.65)	0.028*
Flunitrazepam	6	4224	2.32 (1.01‒5.31)	0.046*
Vonoprazan	5	3978	3.41 (1.38‒8.44)	0.008**

The adjusted reporting odds ratio (aROR) and 95% confidence intervals (95% CI) of microscopic colitis were calculated for sex, age, eBMI and each drug.

*eBMI* estimated body mass index.

***p < 0.001, **p < 0.01, *p < 0.05, p: statistical significance obtained in multiple logistic regression analysis.

**Table 3:** Multiple logistic regression analysis of microscopic colitis using variables of drug and patient background for each sex.

	Cases	Non-cases	aROR (95% CI)	p value
Females	n = 114	n = 114,463		
Age ≥ 60 years	106	71,545	4.45 (2.16‒9.19)	< 0.001***
eBMI				
Obese	32	23,121	1.58 (1.03‒2.43)	0.038*
Underweight	22	18,034	1.40 (0.86‒2.29)	0.181
Normal	60	73,308	Ref.	–
Lansoprazole	96	9888	45.33 (27.22‒75.48)	< 0.001***
Aspirin	27	7,370	1.59 (1.02‒2.46)	0.040*
Males	n = 47	n = 132,373		
Age ≥ 60 years	42	92,572	2.34 (0.92‒5.98)	0.075
eBMI				
Obese	4	21,453	0.47 (0.17‒1.31)	0.147
Underweight	6	22,119	0.74 (0.31‒1.75)	0.489
Normal	37	88,801	Ref.	‒
Lansoprazole	32	12,499	15.32 (8.12‒28.91)	< 0.001***
Aspirin	19	13,208	2.80 (1.52‒5.14)	< 0.001***

The adjusted reporting odds ratio (aROR) and 95% confidence intervals (95% CI) of microscopic colitis were calculated for sex, age, eBMI, lansoprazole and aspirin.

*eBMI* estimated body mass index.

***p < 0.001, *p < 0.05, p: statistical significance obtained in multiple logistic regression analysis.

## Discussion

In the present study, we analyzed the time to onset of MC and evaluated the association between MC and each drug and patient background. Our results suggest that the onset duration of MC was 1‒5 months in lansoprazole users and 2‒33 months in aspirin users. Furthermore, our results suggest that MC was associated with nicorandil, as well as with lansoprazole and aspirin. Although the severity of MC was mild in many cases, even mild MC may decrease the patient’s quality of life. It is important to discontinue the causative drugs for the improvement of symptoms.

A previous study reported that the mean duration of drug exposure before the start of diarrhea in MC patients was very long (15‒60 months) and that of lansoprazole was 4 months^31^. Another study found that the proportion of long-term users of NSAIDs (> 6 months) was higher in the MC group than in patients with irritable bowel syndrome (IBS) or colonic diverticular disease: in addition the mean ± standard deviation duration of onset of MC was 5.5 ± 4.4 years and ranged from 0.5‒15 years^32^. In addition, the duration of exposure to NSAIDs or PPIs for 4–12 months increased the risk of MC^33^. The median onset duration for lansoprazole and aspirin was shorter in the present study than in previous studies^31–33^; additionally, in the present study, the duration of lansoprazole tended to be shorter than that of aspirin. These results suggest that the long-term use of lansoprazole and aspirin may induce the onset of MC; therefore, it is important to identify the starting date of each drug.

A multiple logistic regression analysis in all of the cases revealed that MC was significantly associated with female sex and age ≥ 60 years. The subset analysis revealed that MC was significantly associated with age and obesity in females. The risk of MC has been observed to be higher in females aged > 50 years^3,10–13^, and our results are consistent with these previous studies. Previous studies have reported that obesity was associated with a lower risk of MC^34,35^; however, the present results suggest that obesity was positively associated with MC in females. Regarding underlying diseases, in Japan, CC is most commonly associated with hypertension and reflux esophagitis, whereas an association with autoimmune disorders has been reported in Europe and the United States^7,8^. In addition, the proportion of drug-induced MC was reported to be higher in Japan than in Europe and the United States^6^. Obesity is a risk factor for lifestyle diseases (such as hypertension), and the treatment of lifestyle diseases commonly requires patients to take many drugs, which may have correspondingly induced the onset of MC in Japanese patients. However, there is a lack of data regarding the association between MC and BMI in Japanese patients, and further research is necessary.

In addition to the association of MC with the female sex and older age, as mentioned above, associations with lansoprazole, aspirin, diclofenac and rabeprazole have also been reported. Lucendo et al. assessed the levels of probability at which different drugs can trigger MC^20^ based on the study of Beaugerie and Pardi^15^. In agreement with the present results, they observed the highest likelihood for aspirin, NSAIDs, and lansoprazole^20^. The PPIs evaluated by Lucendo et al. also included omeprazole and esomeprazole^20^. Previous studies have postulated several underlying mechanisms of PPI-induced MC, including their effects on tight junction functionality resulting in increased intestinal permeability^17^, alterations of intestinal microbiota^36^, and colonic dysbiosis^37^ related to acid suppression. These effects were also observed in users of other PPIs. In Japanese patients with MC, a higher proportion used lansoprazole than other PPIs^6,38^. However, a drug-class effect cannot be excluded and rabeprazole and vonoprazan (which is a potassium-competitive acid blocker) can also induce MC. Similarly, NSAIDs can cause MC, and this mechanism has been suggested to impair prostaglandin synthesis, thus resulting in increased intestinal permeability^33^. Increasing intestinal permeability induced by concomitant PPI and NSAID uses may be the underlying mechanism of MC.

The multiple logistic regression analysis revealed that nicorandil and flunitrazepam were also associated with MC. Nicorandil, which is a potassium channel opener and nitric oxide (NO) donor^39^, is commonly used for the treatment of angina pectoris. Although nicorandil can induce headaches with mild to moderate severity, it was generally well tolerated in clinical trials^40^. However, nicorandil may also cause gastrointestinal ulcers, fistulation^41^, and oral ulceration^42^. The release of inflammatory NO in the intestinal mucosa has been postulated as the mechanism of fistulation^43^. The abnormal metabolism of NO is considered to be one of the mechanisms of diarrhea in MC^44^; therefore, nicorandil may cause MC. In addition, β-blockers and calcium channel blockers are also used for the treatment of angina pectoris, and a disproportionate occurrence of MC was observed in our study in users of these drugs (Supplementary Tables S2). Thus, we cannot exclude the hypothesis that the condition of angina pectoris can cause MC. MC was also associated with flunitrazepam, although we were unable to determine a plausible biological mechanism. Moreover, magnesium oxide was observed to be negatively associated with MC. This scenario is due to magnesium oxide being commonly used as an osmotic purgative and is rarely used in MC patients presenting with diarrhea symptoms. The association between MC and magnesium oxide remains unclear, and further research is needed.

Our study had several limitations. First, the outcomes of the study using the JADER database may be affected by the lack of a denominator, over- or underreporting, and various biases, such as a competition bias and notoriety bias. The onset duration of MC may be shorter than the actual duration because reporters generally recognize recently started drugs in the adverse drug events report. Additionally, we could not investigate the association between MC and a dose-related effect of drugs because there is considerable missing information about drug dosages in the JADER database. A previous study reported that the risk of developing MC did not differ by a daily dose of NSAIDs, PPIs, SSRIs, and statins^20^. However, the dose-related effects of other drugs are possibly associated with the onset of MC, and further research is needed. Second, the clinical symptoms of MC are similar to those of IBS^45^, and patients with MC could have been misdiagnosed as having IBS. In addition, we could not classify CC and LC because adverse events were reported by using PT. These limitations could have affected the results of our study. Third, the stepwise method has been used to detect the previously unknown adverse drug events in previous studies^46–49^, but this method should be considered the limitation that the real explanatory variables that have causal effects on the objective variable may not be coincidentally statistical significance, while nuisance variables may be coincidentally significant^50^. Furthermore, a study using the JADER database cannot evaluate a true risk and provides a hypothesis as a starting point for exploratory analyses^51^. Our findings should be interpreted with caution due to the in silico approach that was used; thus, the results cannot provide causal inference between MC and each drug. A prospective clinical trial is needed to further evaluate these results. Nevertheless, this is the first study in Japan to evaluate the time-to-onset profile of MC and the pharmacoepidemiology of MC by using the JADER database.

## Conclusions

In the present study, we analyzed the time-to-onset profile of MC and evaluated the association between MC and each drug and patient background by using the JADER database. Our results suggest that the onset duration of MC was 1‒5 months in lansoprazole users and 2‒33 months in aspirin users. A multiple logistic regression analysis revealed that MC was significantly associated with the female sex, age ≥ 60 years, and drugs including lansoprazole, aspirin, magnesium oxide, nicorandil, diclofenac, rabeprazole, flunitrazepam and vonoprazan. A subset analysis revealed that MC was positively associated with obesity in females. Our study used an in silico approach and cannot provide causal inference between MC and each drug; however, MC was associated with nicorandil as well as with lansoprazole and aspirin. These findings need to be evaluated in cohort studies and long-term clinical investigations.

## Supplementary Information

Supplementary Tables.

## Data Availability

The datasets generated during and/or analyzed during the current study are available in the JADER database, https://www.pmda.go.jp/safety/info-services/drugs/adr-info/suspected-adr/0004.html.
