# Supplementary material for: Adverse event profiles of microscopic colitis in the Japanese Adverse Drug Event Report (JADER) database
Source: Sci Rep. 2022 Oct 21;12:17652. doi: 10.1038/s41598-022-22257-2 (PMC9587040; doi:10.1038/s41598-022-22257-2)
Supplement: Supplementary file 1 — Supplementary Tables. [file 41598_2022_22257_MOESM1_ESM.pdf]

Supplementary information

Adverse event profiles of microscopic colitis in the Japanese Adverse Drug Event Report (JADER) database

Authors: Kaito Yamashiro [1,2], Mika Jouta [2], Kouichi Hosomi [3], Satoshi Yokoyama [3], Yuu Ozaki [2], Atsushi Hirata [2], Fumihiko Ogata [1], Takehiro Nakamura [1], Shigeharu Tanei [4], and Naohito Kawasaki [*,1]

[1] *Laboratory of Public Health, Faculty of Pharmacy, Kindai University, 3–4–1, Kowakae, Higashi-Osaka, Osaka 577–8502, Japan*
[2] *Department of Pharmacy, Kindai University Nara Hospital, 1248–1, Otodacho, Ikoma, Nara 630–0293, Japan*
[3] *Division of Drug Informatics, Faculty of Pharmacy, Kindai University, 3–4–1 Kowakae, Higashi-Osaka, Osaka 577–8502, Japan*
[4] *Faculty of Pharmaceutical Sciences, Nihon Pharmaceutical University, 10281 Komuro, Ina-machi, Kitaadachi-gun, Saitama 362–0806, Japan*

*Corresponding Author: Naohito Kawasaki, Laboratory of Public Health, Faculty of Pharmacy, Kindai University, 3–4–1, Kowakae, Higashi-Osaka, Osaka 577–8502, Japan
Email: Kawasaki@phar.kindai.ac.jp; Telephone number: +81 6–4307–4012

This document contains:
Supplementary Table S1
The variance inflation factors values of the variables in the final multiple logistic regression model
Supplementary Table S2
Number of cases and non-cases of microscopic colitis, and crude reporting odds ratio, in the most common therapeutic categories
Supplementary Table S3
Number of cases and non-cases of microscopic colitis, and crude reporting odds ratio, of microscopic colitis in other drugs

Supplementary Table S1. The variance inflation factors values of the variables in the final multiple logistic regression model

	**Variance inflation factors**		
	**All cases**	**Female cases**	**Male cases**
Sex (female)	1.013	N/A	N/A
Age ≥ 60 years	1.041	1.024	1.026
eBMI			
Obese	1.049	1.053	1.045
Underweight	1.050	1.051	1.052
Normal	N/A	N/A	N/A
Lansoprazole	1.044	1.021	1.028
Aspirin	1.102	1.028	1.040
Magnesium oxide	1.035	N/A	N/A
Nicorandil	1.064	N/A	N/A
Diclofenac	1.004	N/A	N/A
Rabeprazole	1.017	N/A	N/A
Flunitrazepam	1.007	N/A	N/A
Vonoprazan	1.006	N/A	N/A

N/A: not available.

Supplementary Table S2. Number of cases and non-cases of microscopic colitis, and crude reporting odds ratio, in the most common therapeutic categories

	**Cases n = 161**	**Non-cases n = 246,836**	**cROR (95% CI)**
PPIs			
Lansoprazole	128	22,387	38.89 (26.52–57.03)
Rabeprazole	8	10,399	1.19 (0.58–2.42)
Esomeprazole	6	8,825	1.04 (0.46–2.36)
Vonoprazan	5	3,978	1.96 (0.80–4.77)
NSAIDs			
Aspirin	46	20,578	4.40 (3.12–6.19)
Loxoprofen	16	19,087	1.32 (0.79–2.21)
Diclofenac	11	5,544	3.19 (1.73–5.89)
Celecoxib	6	5,409	1.73 (0.76–3.91)
ACE-Is/ARBs			
Candesartan	14	8,704	2.61 (1.51–4.51)
Olmesartan	13	6,527	3.23 (1.83–5.70)
Valsartan	11	8,001	2.19 (1.19–4.04)
Losartan	6	4,441	2.12 (0.93–4.78)
Enalapril	7	3,850	2.87 (1.34–6.12)
Statins			
Rosuvastatin	14	7,128	3.20 (1.85–5.54)
Atorvastatin	10	8,701	1.81 (0.96–3.44)
Pravastatin	8	4,902	2.58 (1.27–5.26)
β-blockers			
Carvedilol	14	7,700	2.96 (1.71–5.12)
Bisoprolol	12	6,276	3.09 (1.71–5.56)
Bisphosphonates			
Alendronic acid	13	5,260	4.03 (2.29–7.12)
Risedronic acid	5	2,773	2.82 (1.16–6.88)
Calcium channel blockers			
Amlodipine	28	26,892	1.72 (1.15–2.59)
Nifedipine	8	8,381	1.49 (0.73–3.03)
Diltiazem	6	2,557	3.70 (1.63–8.37)
Benidipine	6	2,896	3.26 (1.44–7.38)

cROR: crude reporting odds ratio; CI: confidence interval.

Supplementary Table S3. Number of cases and non-cases of microscopic colitis, and crude reporting odds ratio, of microscopic colitis in other drugs

	Cases<br>n = 161	Non-cases<br>n = 246,836	cROR (95% CI)
Furosemide	21	19,267	1.77 (1.12–2.80)
Nicorandil	15	4,219	5.91 (3.47–10.06)
Allopurinol	15	9,755	2.50 (1.47–4.25)
Magnesium oxide	15	25,972	0.87 (0.51–1.49)
Etizolam	14	6,785	3.37 (1.95–5.83)
Rebamipide	13	14,897	1.37 (0.78–2.41)
Clopidogrel	12	5,930	3.27 (1.82–5.89)
Ferrous citrate	12	5,302	3.67 (2.04–6.61)
Mecobalamin	12	8,240	2.33 (1.29–4.20)
Brotizolam	10	8,576	1.84 (0.97–3.49)
Teprenone	10	6,842	2.32 (1.22–4.41)
Prednisolone	10	25,380	0.58 (0.30–1.10)
Ursodeoxycholic acid	9	9,343	1.51 (0.77–2.95)
Alfacalcidol	9	7,871	1.80 (0.92–3.52)
Cilostazol	8	3,663	3.47 (1.70–7.07)
Warfarin	8	8,533	1.46 (0.72–2.97)
Zolpidem	8	7,352	1.70 (0.84–3.47)
Mosapride	7	5,245	2.09 (0.98–4.47)
Febuxostat	6	5,803	1.61 (0.71–3.64)
Spironolactone	6	8,167	1.13 (0.50–2.56)
Flunitrazepam	6	4,224	2.22 (0.98–5.03)
Loperamide	6	2,223	4.26 (1.88–9.64)
Isosorbide nitrate	5	3,049	2.56 (1.05–6.25)
Apixaban	5	2,931	2.67 (1.09–6.50)
Triazolam	5	2,636	2.97 (1.22–7.24)
Albumin tannate	5	603	13.09 (5.35–32.00)
Tocopherol nicotinate	5	1,722	4.56 (1.87–11.13)
Famotidine	5	19,799	0.37 (0.15–0.90)

cROR: crude reporting odds ratio; CI: confidence interval.
